# Supplementary material for: High-Frequency Tails in Spectral Densities
Source: J Phys Chem A. 2025 Apr 4;129(15):3587–96. doi: 10.1021/acs.jpca.5c00943 (PMC12010327; doi:10.1021/acs.jpca.5c00943)
Supplement: Supplementary file 1 — jp5c00943_si_001.pdf [file jp5c00943_si_001.pdf]

Supporting Information: High-Frequency Tails in Spectral Densities

Roman Korol,[*,†] Xinxian Chen,[†] and Ignacio Franco[*,†]

†*Department of Chemistry, University of Rochester, Rochester, New York 14627, USA*

‡*Department of Physics, University of Rochester, Rochester, New York 14627, USA*

E-mail: roman.korol@rochester.edu; ignacio.franco@rochester.edu

1 2-peak spectral density functions

The normalization constants for the two-peak spectral density basis functions are presented below. We show the simulations only for the Ohmic [Eq. (S1)] and the superohmic [Eq. (S2)] cases in Fig. 3.

$$\Lambda_{1,k} = \frac{\Lambda_{2p,k} \prod_{i=\{k_1,k_2\}} \gamma_i(\omega_i^2 + \gamma_i^2)(\omega_i - \omega_j + \gamma_i)}{\sum_{i=\{k_1,k_2\}, j\neq i} \gamma_i \left(\gamma_i^2 + 4\gamma_j^2 + \omega_i^2\right)} \tag{S1}$$

$$\Lambda_{3,k} = \frac{\Lambda_{2p,k} \prod_{i=\{k_1,k_2\}} \gamma_i}{\sum_{i=\{k_1,k_2\}} \gamma_i} \tag{S2}$$

$$\Lambda_{5,k} = \frac{\Lambda_{2p,k} \prod_{i=\{k_1,k_2\}} \gamma_i}{\sum_{i=\{k_1,k_2\}, j\neq i} \gamma_i(\omega_j^2 + \gamma_j^2)} \tag{S3}$$

$$\Lambda_{7,k} = \frac{\Lambda_{2p,k} \prod_{i=\{k_1,k_2\}} \gamma_i}{\sum_{i=\{k_1,k_2\}, j\neq i} \gamma_i \left(4\gamma_i^2\gamma_j^2 + 4\gamma_j^2\omega_j^2 + (\omega_j^2 + \gamma_j^2)^2\right)} \tag{S4}$$

$$\Lambda_{2p,k} = \frac{4(\lambda_{k_1} + \lambda_{k_2})}{\pi} \left((\omega_{k_1} + \omega_{k_2})^2 + (\gamma_{k_1} - \gamma_{k_2})^2\right) \left((\omega_{k_1} - \omega_{k_2})^2 + (\gamma_{k_1} + \gamma_{k_2})^2\right) \tag{S5}$$

To set the peak widths in Table 1 we work backwards from the functional form of the Ohmic 2-peak functions [Eq. (20)] to ensure that the resulting spectral densities from all five basis function choices are similar with a realistic choice of widths. We pair up the eight peaks creating pairs (1,2), (3,4) etc., such that the two peaks in each pair do not have significant overlap and also have similar reorganization energy parameter (λ_k). We then set the peak widths of 10 cm^{-1} for the four odd index peaks. The widths for the other four peaks (with even indices) are set such that the reorganization energies of each individual peak of the Ohmic 2-peak SD functions match the reorganization energy parameters λ_k in Table 1.

2 Derivation of tail adjustment

We derive Eqs. (23) and (24) assuming both the source and target populations follow Lindblad equation.[S61] In this case the population of excited electronic state changes follows the

rate equation:

$$\dot{p}_1(t) = \gamma_- p_0(t) - \gamma_+ p_1(t), \tag{S6}$$

where γ_- and γ_+ are the (constant) rates of absorption and total (stimulated plus spontaneous) emission given in terms of spectral density as follows:

$$\gamma_- = \gamma(-\Omega) = \frac{2\pi\alpha_x^2 J(\Omega)}{e^{\beta\hbar\Omega} - 1} = e^{-\beta\hbar\Omega}\gamma_+ \tag{S7}$$

$$\gamma_+ = \gamma(+\Omega) = \frac{2\pi\alpha_x^2 J(\Omega)}{1 - e^{-\beta\hbar\Omega}} = 2\pi\alpha_x^2 J(\Omega)\left[1 + \frac{1}{e^{\beta\hbar\Omega} - 1}\right]. \tag{S8}$$

After elimination of $p_0(t)$ from Eq. (S6) using $p_0(t) + p_1(t) = 1$ (i.e., ground and excited electronic state populations add up to 1), the equation becomes

$$\dot{p}_1(t) = \gamma_-[1 - p_1(t)] - \gamma_+ p_1(t) = \gamma_- - \gamma p_1(t), \tag{S9}$$

where $\gamma = \gamma_- + \gamma_+$. Taking the following anzatz:

$$p_1(t) = ce^{-bt} + d \tag{S10}$$

with c set by initial condition as $c = p_1(0) - d$. Plugging it into Eq. (S9):

$$-bce^{-bt} = \gamma_- - \gamma ce^{-bt} - \gamma d,$$

so $b = \gamma$ and $d = \frac{\gamma_-}{\gamma} = \frac{e^{-\beta\hbar\Omega}}{1+e^{-\beta\hbar\Omega}} = p_1(\infty) = p_1^{eq}$. Thus,

$$p_1(t) = [p_1^0 - p_1^{eq}]e^{-\gamma t} + p_1^{eq}, \tag{S11}$$

where $p_1^0 = p_1(0)$ is the initial population of the excited state.

To derive Eq. (23) we write Eq. (S11) for both source and target, keeping in mind that initial and final excited state populations of source and target are equal, i.e. $p_{\text{target}}^0 = p_{\text{source}}^0 =$

p_0 and $p^{eq}_{\text{target}} = p^{eq}_{\text{source}} = p_{eq}$

$$\begin{aligned} p_{\text{target}}(t) &= [p^0_{\text{target}} - p^{eq}_{\text{target}}]e^{-\gamma_{\text{target}}t} + p^{eq}_{\text{target}} \\ &= [p_0 - p_{eq}]e^{-\gamma_{\text{target}}t} + p_{eq} \\ &= p_{\text{source}}(t)e^{-[\gamma_{\text{target}}-\gamma_{\text{source}}]t} + p_{eq}\left[1 - e^{-[\gamma_{\text{target}}-\gamma_{\text{source}}]t}\right] \\ &= p_{eq} + [p_{\text{source}}(t) - p_{eq}]\, e^{-[\gamma_{\text{target}}-\gamma_{\text{source}}]t} \end{aligned} \tag{S12}$$

Finally, Eq. (24) follows from Eq. (S7) as

$$\gamma = \gamma_- + \gamma_+ = \gamma_+\left[1 + e^{-\beta\hbar\Omega}\right] = 2\pi\alpha_x^2 J(\Omega)\coth\left(\frac{\beta\hbar\Omega}{2}\right) \tag{S13}$$
